# An example of DNA methylation as a means to quantify stress in wildlife using killer whales

**DOI:** 10.1038/s41598-021-96255-1

**Published:** 2021-08-19

**Authors:** Carla A. Crossman, Lance G. Barrett-Lennard, Timothy R. Frasier

**Affiliations:** 1 Biology Department, Saint Mary’s University, Halifax, NS Canada; 2 Coastal Ocean Research Institute, Ocean Wise Conservation Association, Vancouver, BC Canada

**Keywords:** Conservation biology, DNA methylation

## Abstract

The cumulative effects of non-lethal stressors on the health of biodiversity are a primary concern for conservation, yet difficulties remain regarding their quantification. In mammals, many stressors are processed through a common stress-response pathway, and therefore epigenetic changes in genes of this pathway may provide a powerful tool for quantifying cumulative effects. As a preliminary assessment of this approach, we investigated epigenetic manifestations of stress in two killer whale populations with different levels of exposure to anthropogenic stressors. We used bisulfite amplicon sequencing to compare patterns of DNA methylation at 25 CpG sites found in three genes involved in stress response and identified large differences in the level of methylation at two sites consistent with differential stress exposure between Northern and Southern Resident killer whale populations. DNA methylation patterns could therefore represent a useful method to assess the cumulative effects of non-lethal stressors in wildlife.

## Introduction

Although lethal threats such as directed hunting or incidental mortality (e.g., due to habitat loss or accidental mortality such as bycatch) represent the largest threats to global biodiversity^1–3^, there is increasing recognition that non-lethal threats are compromising the viability of many populations, particularly when experienced in combination and/or when they have synergistic effects^4–6^. For example, the global colony collapse of honey bees and the global declines in amphibian populations have both been attributed to the combined effects of multiple non-lethal threats^7,8^. As a result, there is a growing emphasis on the need to consider the cumulative effects of non-lethal threats in the management and conservation of biodiversity^9–11^.

Many non-lethal threats—including those from anthropogenic activities such as physical disturbance and contaminant exposure—are thought to increase stress on individual animals, with subsequent negative fitness consequences. Despite being well-documented in humans and mice, quantifying the negative fitness consequences of stress in wildlife populations remains a challenge. Stress hormones can be measured in blood, saliva, excrement and hair/feathers^12,13^; however, collection of these samples is difficult with many species and stress hormones often only capture a snapshot of an animal’s recent stress exposure. Quantifying the cumulative effects of multiple stressors poses an additional obstacle, particularly if their effects are synergistic, as meta-analyses often suggest^14^.

One approach that may be particularly informative for understanding the cumulative impacts of stressors is the study of methylation patterns^15,16^, and in particular that of genes involved in stress response. DNA methylation is an epigenetic modification to DNA that occurs primarily at 5'-CpG-3' (cytosine-guanine dinucleotide—CpG) sites^17^. CpG sites are not evenly distributed throughout the genome, but rather are concentrated in ‘CpG islands’ in the promoter region of many genes where they act as important regulators of gene expression^18^. The methylation of some sites in the genome can be influenced by extrinsic factors experienced by individuals including stress exposure, and some may even be non-reversible (i.e., the biological effects of the stressor may last long after the initial stressor is gone)^19–21^.

Stress-related changes in DNA methylation have been detected in genes in the hypothalamic pituitary adrenal (HPA) axis^22–26^—a main pathway regulating stress response in mammals^27^. The HPA axis is regulated by a suite of genes including the brain derived neurotrophic factor (*BDNF*) and the corticotropin releasing factor (*CRF*)^28^. *CRF* initiates the stress response pathway that culminates in glucocorticoids (stress hormones) being released^29^. The cellular responses to these hormones are mediated largely by the glucocorticoid receptor (*NR3C1*)^30^. The complexity of the stress response pathway relies on proper functioning of these and other genes and therefore altered methylation patterns could have significant downstream effects. For example, controlled laboratory experiments on mice and rats have identified changes in methylation patterns in response to stressors in genes in the HPA axis^22–26^, and similar results have been identified in human studies^25,31^. Therefore, analyses of methylation patterns of stress-response genes have the potential to be particularly informative in assessing stress in wildlife populations because these patterns represent the cumulative impacts of the suite of stressors experienced by an individual, and therefore provide a method to quantify their cumulative and/or synergistic effects.

The Southern Resident population of killer whales (*Orcinus orca*) in the Northeast Pacific is an endangered population (N = 74)^32^ whose main identified threats are the cumulative impacts of multiple non-lethal stressors (e.g., underwater noise, vessel disturbance, food availability and toxic contaminants)^33^. To the north is the closely related and more robust population of Northern Resident killer whales (N ≈ 300)^34^. Northern and Southern Resident killer whale populations have similar dietary preferences and inhabit adjacent, slightly overlapping ranges^35,36^. The Southern Resident killer whale range has greater overlap with heavily inhabited coastlines and transited waterways compared to Northern Residents and as such the population experiences higher levels of exposure to anthropogenic stressors. We predict that the differential exposure to stressors will manifest themselves as differences in the amount of DNA methylation at CpG sites in genes involved in stress response.

Understanding the combined biological impacts of these non-lethal threats on individual fitness might therefore best be explored by examination of epigenetic differences in genes related to stress. As a step towards this goal, we identified the methylation patterns of stress-related genes in individual Southern Resident killer whales and compared them to those in Northern Resident individuals.

## Results

**Figure 1:**
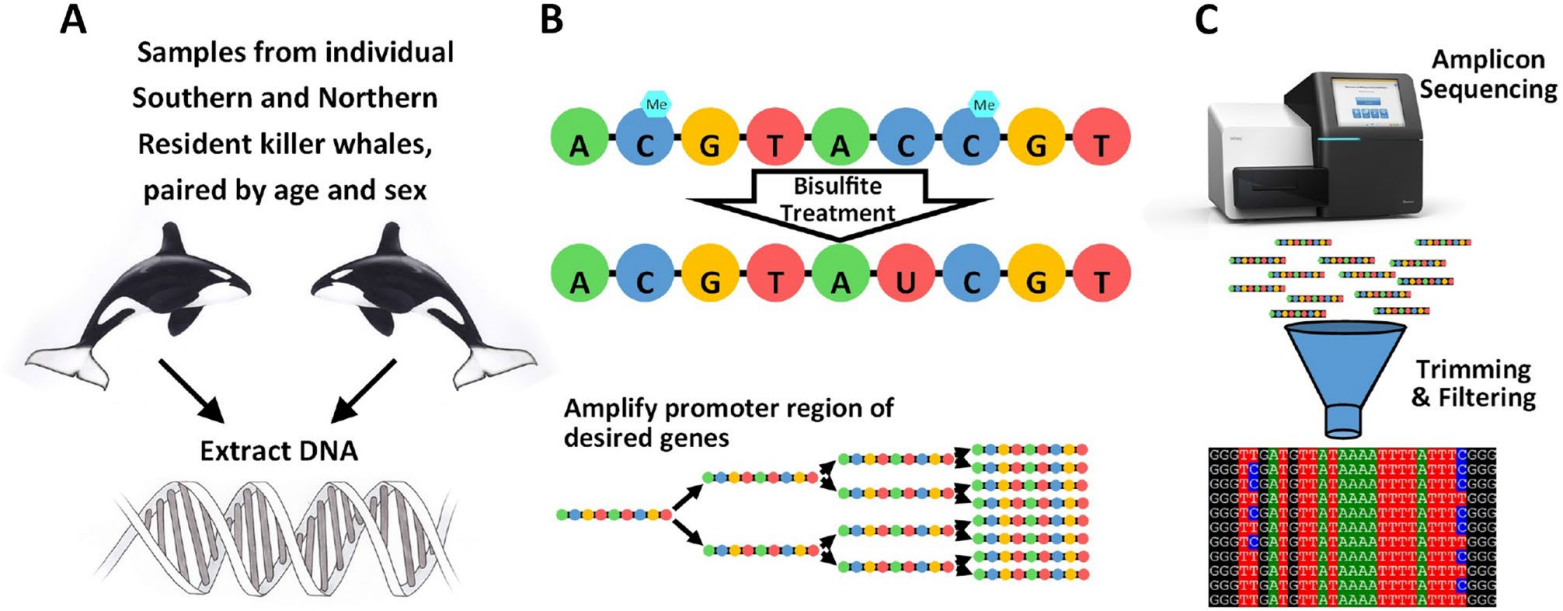
Summary of methods to generate percent of methylation at each CpG site. **(A)** Biopsy samples were collected from Northern and Southern Resident killer whales and DNA was extracted. **(B)** DNA underwent a bisulfite treatment that converts unmethylated cytosines to uracil. This transformed DNA was used as the template for amplification of four desired genes. **(C)** Pooled libraries were sequenced on a MiSeq. Output reads were trimmed, filtered, and aligned to reference sequences and percent methylation at each CpG site was calculated for each individual. See supplementary material for additional detailed methods. Image of killer whales provided by Dusan Postolovic and of MiSeq courtesy of Illumina, Inc.

**Figure 2:**
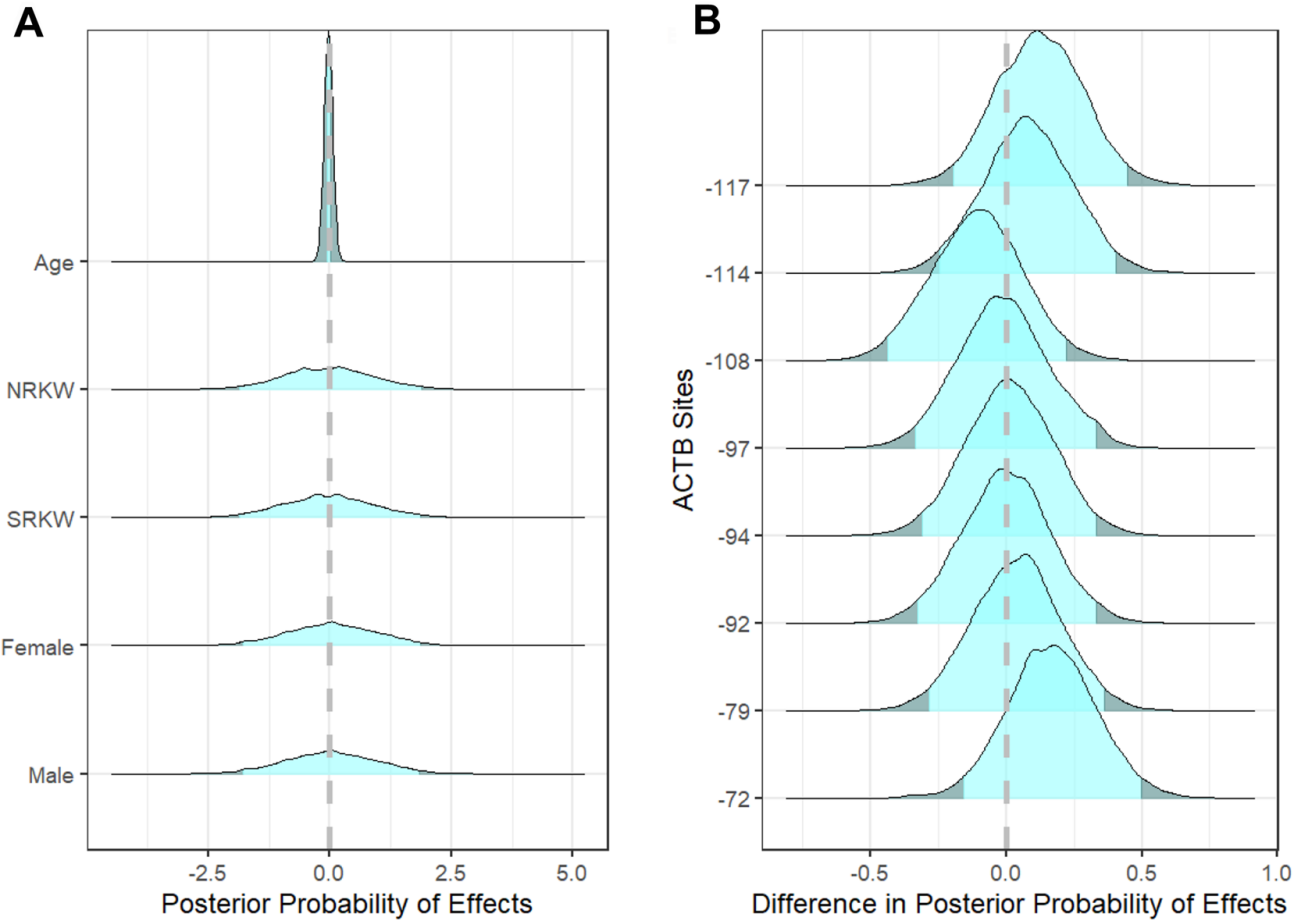
**(A)** The independent effects of age, population (Northern and Southern Resident killer whales: NRKW and SRKW) and sex on percent methylation of examined CpG sites showed little effect of total methylation patterns. **(B)** The control gene ACTB showed little to no difference in methylation patterns between Southern and Northern Residents. Y-axis is the number of base pairs prior to the transcriptional start site (TSS), hence the negative values. Positive X-values indicate hypermethylation in Southern Residents relative to Northern Residents and negative values indicate hypomethylation. The distribution falling outside the 95% highest density interval is indicated with the darker shading.

Using amplicon sequencing of sodium bisulfite treated DNA extracted from biopsy samples of skin from free swimming killer whales, we identified percent methylation for CpG sites in the promoter region of three genes known to be key players in stress response: *BDNF*, *CRF* and *NR3C1*; as well as *ACTB* (β-actin)—a gene commonly used as a control as it regulates components of the cytoskeleton^37^ (Figure S1). We used a Bayesian regression model to tease apart the relative effects of individual, age, sex, population, and CpG site on methylation patterns (Fig. 1). Neither age nor sex had appreciable effects on methylation patterns, with the mean of the posterior distributions falling close to zero (Fig. 2a), confirming that methylation of the targeted CpG sites does not change with age or under the different hormonal exposures experienced across the two sexes. Thus, any differences in methylation patterns should be due to differences in the levels of external stressors. To compare the relative effects of being in a given population on the level of DNA methylation at a given site, we took the difference in the posterior probability distributions at each site between populations. If the population of origin had little to no impact on the amount of methylation, the distribution will centre around zero, whereas a positive distribution would indicate hypermethylation in Southern Residents compared to Northern Residents and a negative distribution would be indicative of hypomethylation. As expected, CpG sites within the promoter of the control gene (*ACTB*) did not show different degrees of methylation across the two populations (Fig. 2b).

**Figure 3:**
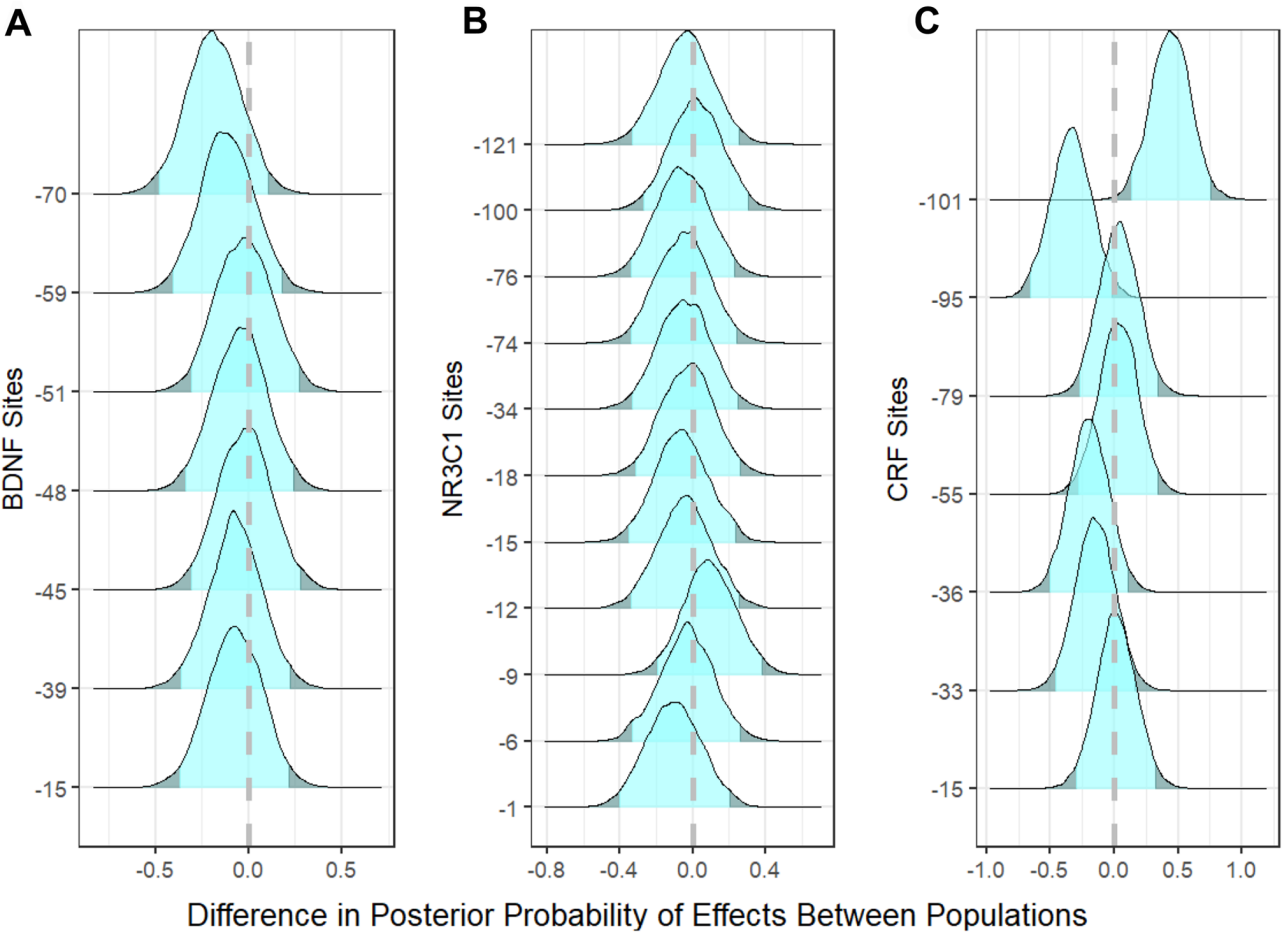
Differences in the posterior probability distributions of estimated effects between Northern and Southern Resident killer whale populations. Included are estimates for each CpG site identified by their distance from the TSS for **(A)** BDNF, **(B)** NR3C1 and **(C)** CRF. Positive values indicate Southern Residents are hypermethylated compared to Northern Residents and negative values indicate hypomethylation in Southern Residents compared to Northern Residents. The distribution falling outside the 95% highest density interval is indicated with the darker shading.

Of the genes examined, the CpG sites in the promoter regions of two (*BDNF* and *NR3C1*) showed similar degrees of methylation across populations (Fig. 3a,b). However, site-specific differences in methylation patterns were seen in the promoter region of *CRF* (Fig. 3c). The largest differences were in position −101 that is hypermethylated in Southern Resident individuals relative to the Northern Residents and in position −95 that is hypomethylated in Southern relative to North Residents (Fig. 3c).

## Discussion

Methylation patterns at stress response genes differed between Northern and Southern Resident killer whales that could not be explained by age or sex and are likely due to differences in stress exposure. Many studies have demonstrated that methylation patterns at some CpG sites change with age in very predictable ways and can be used in age-predictive models^38^. We chose a paired sample approach to help elucidate the possible effect of population, while also accounting for potential effects of age and sex. Our results suggest the CpG sites in the genes targeted in our study are not affected by age or sex, but rather methylation difference reflect differences in the environments experienced by Resident killer whales.

While genetically distinct^39^, these two populations of killer whales are remarkably similar. The have the same dietary preferences, adjacent but overlapping distributions, and very similar physical appearances and acoustic behaviours^35,36^. One of the primary differences in the environments experienced by these two populations is the degree of exposure to anthropogenic stress. Southern Residents spend much of the year in the interior waters near the busy port cities of Vancouver and Victoria, Canada; and Seattle, USA, and therefore the whales in this area are exposed to high levels of pollution, heavy vessel traffic from recreational boaters, a large commercial whale watching fleet, and large cargo vessels causing both acoustic and physical disturbance^33^. In contrast, Northern Residents inhabit quieter, more pristine waters to the north with less commercial and recreational vessel presence.

The patterns we identified in *CRF* (hypermethylation at position −101 and hypomethylation at position −95) have also been observed in some tissues of rats exposed to chronic variable mild stress^26^ and since the *CRF* promoter region in killer whales has over 95% similarity with other mammalian species (Figure S2), this suggests that they serve the same functions across species. Additionally, altered methylation patterns in response to stress have previously been identified at position −101 in other species, but the direction of these methylation changes has differed between tissue types and studies^22,23^.

Only one of these two sites (position −101) falls within a previously identified transcription factor binding region: that for the aryl hydrocarbon receptor (*Ahr*)^40,41^. The *Ahr* binding region (position −101) is particularly interesting because, in addition to being involved in stress response, aryl hydrocarbon receptors are also involved in the metabolism of polycyclic aromatic hydrocarbons (PAHs)^42^, contaminants to which killer whales are frequently exposed but which do not tend to bioaccumulate and exposure is therefore difficult to assess^43^.

Current practices for understanding the cumulative effects of multiple stressors on individuals rely on models that incorporate estimated effects of independent stressors and make assumptions about how these stressors interact and are experienced by the individual^44,45^. The potential to use changes in the amount of methylation at stress-related genes within a population over time or between populations, may therefore represent a stronger approach for understanding the true biological impact and fitness consequences of multiple stressors experienced by individuals. This study represents the first step in demonstrating the application of this method for a mammalian population, which may prove to be an effective long term monitoring tool for wildlife populations.

For Southern Residents, the biological implications of these changes in methylation are yet to be determined. Further investigations into the biological or physiological implications of these methylation differences are needed, but our results suggest that long-term monitoring of levels of methylation in stress-related genes may provide a useful tool for quantifying the synergistic effects of multiple stressors, and help track the effectiveness of threat mitigation efforts on reducing stress on individual killer whales.

The cumulative and synergistic effects of anthropogenic-induced stress are likely influencing a wide range of wildlife populations, but quantifying such effects has not yet been possible. Assessing DNA methylation of genes involved in stress response may represent a promising option, as suggested by the application of this approach to critically endangered Southern Resident killer whales. Although we do not yet know the downstream biological implications of the differential methylation patterns observed, this represents a crucial first step in making such quantification possible.

## Methods

### Library preparation and sequencing

**Figure 4:**
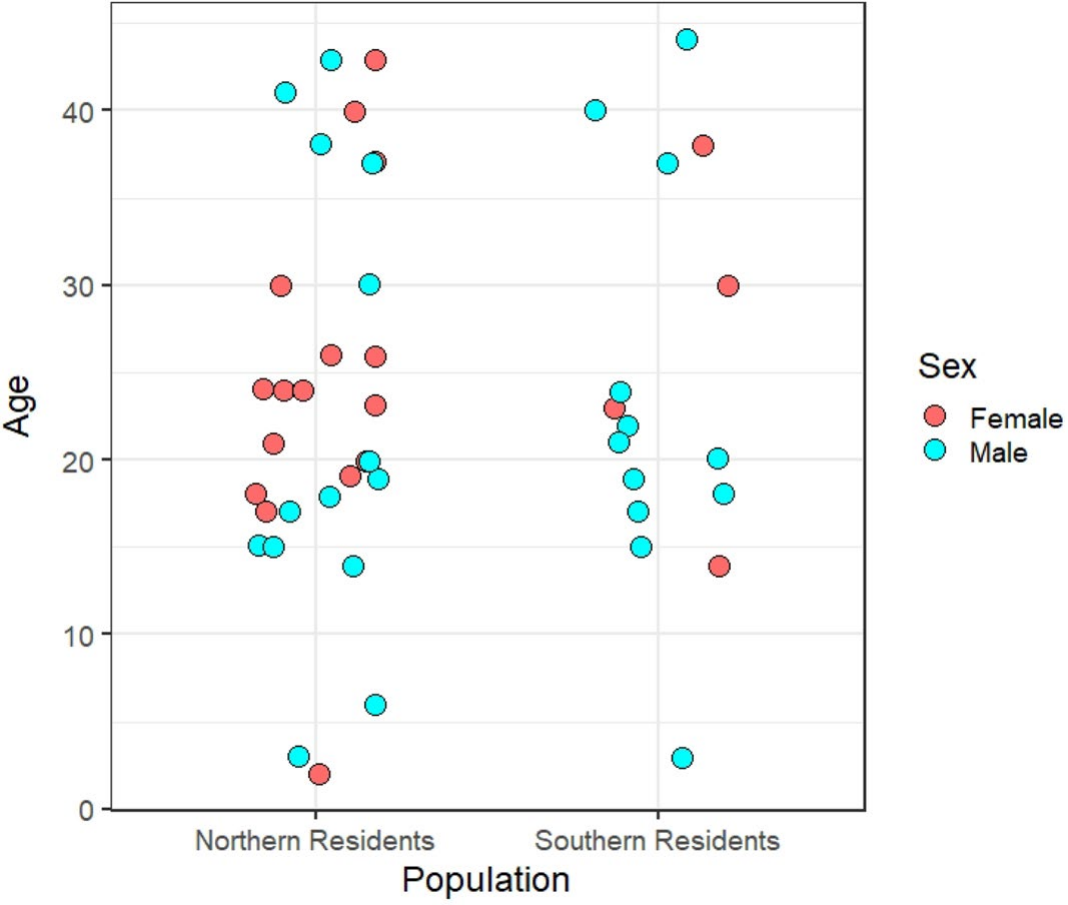
The distribution of age and sex of individuals in each population used in this study.

DNA samples for this study are part of a long-term tissue collection not collected explicitly for this study. Skin samples used were collected from free-swimming whales for previous work using a pneumatic dart system^46^, except for one sample that was collected from a freshly deceased carcass. During biopsy sampling, biopsy darts collect a small piece of skin from the dorsal surface of the animal, near its dorsal fin. This biopsy method is consistent between populations. A paired sample approach was implemented when selecting which of the existing samples to use so that for each Southern Resident sample with sufficient high-quality DNA (n = 17), one or two Northern Residents of the same sex and similar age were included (n = 30) (Fig. 4).

The DNA was previously extracted using standard phenol:chloroform methods^47^ using the skin adjacent to the skin-blubber interface of each sample. 400 ng of DNA from each individual was subjected to sodium bisulfite conversion using Epitect Bisulfite Kits (Qiagen). This process converted unmethylated cytosines (C) in CpG sites to uracil (U), whereas methylated cytosines in CpG sites remained C.

We identified three genes in the HPA axis and its regulation that had demonstrated methylation changes in response to stressors in controlled studies of laboratory animals: brain derived neurotropic factor (*BDNF*), corticotropin releasing factor (*CRF*) and glucocorticoid receptor (*NR3C1*) as well as a common control gene, β-actin (*ACTB*) whose methylation patterns should not be influenced by age, sex, or exposure to external stressors^37^, rather interpopulation differences here could represent genetic differences in methylation/demethylation enzymes. We identified reference sequences for the promoter region of each of these genes from the published killer whale genome (GenBank accession GCA_000331955.1). We did an in-silico sodium bisulfite conversion of our reference sequences and designed primers accounting for the DNA being single stranded and non-complementary post bisulfite treatment, avoiding CpG sites within the primer sequence if possible (see supplementary material). We performed Sanger sequencing to test that: (i) we had amplified the correct region, and (ii) the resulting sequences would be absent of cytosine except in potentially methylated sites.

Each bisulfite-treated sample was amplified using each primer pair. Our subsequent library preparation protocol was a modified version of that available for 16S sequencing^48^.

We cleaned our PCR products with Ampure XP beads using bead:sample ratios optimized for each primer set and final elution volumes adjusted for each sample as determined by concentration on a pre-cleanup agarose gel. We performed an indexing PCR to bind sample-specific indexes using the Nextera xt Index kit (Illumina) and cleaned the products. The final libraries were quantified, pooled and sequenced on an Illumina MiSeq 250 × 250 PE run at the BRC Sequencing Core at the University of British Columbia (Vancouver, Canada).

### Processing amplicon sequences

Each index was unique to a single individual but included amplicons from multiple loci. Separating loci and trimming primer sequences was performed in CUTADAPT 2.6 with an allowable error rate of 0.2^49^. Leading and trailing bases with a Q < 20 were trimmed and reads were trimmed when the mean quality in a 5-base sliding window fell below Q = 30 in TRIMMOMATIC 0.36^50^. Paired reads were merged using FLASH 1.2.11^51^. Aligned and indexed bam files were compiled using BWA 0.7.17-r1188 and SAMTOOLS 1.7^52,53^ and aligned against the reference genes used to design our primers.

Despite normalizing our library concentrations prior to sequencing, we had differential amplification and sequencing success of each locus resulting in an uneven distribution of reads across loci. As a result, we determined minimum read depth thresholds independently for each locus. Our goal was to identify percent methylation at each site and therefore wanted to optimize read depth to increase confidence in our measures of percent methylation while still maintaining relatively consistent certainty within each locus. We visualized read depths using histograms of read depth across samples and determined the minimum threshold for each locus as:* ACTB*—10 000,* BDNF*—20 000,* CRF*—100,* NR3C1*—500 (table S2 and figure S3). Individuals were excluded from the analysis if a minimum read depth threshold was not met. Due to low read depths of merged pair-end reads for *NR3C1*, forward unpaired reads were also trimmed and included in the aligned bam file to increase read depth.

### Analyses

Using the reference sequence for each locus, we identified potentially methylated sites (CpG sites). Methylated CpG sites in our reads would have escaped bisulfite conversion and remained as C bases whereas unmethylated CpG sites would be represented by Ts. For every individual at each gene, we calculated the proportion of C/T base calls as a metric of percent methylation at each CpG site.

We built a Bayesian regression model using the percent methylation of each site as the predicted variable where individual, age, sex, population and CpG site were the predictor variables, as well as the interaction between specific CpG sites and population. We also allowed for different standard deviations in each population. We built similar models investigating other pairwise interactions and none had an effect on our results, so we left them out of our final model. (See supplementary material for additional details of the model and its performance).

To better visualize and understand the differences between populations, we plotted the difference in the posterior probabilities of percent methylation at each site calculated as that from Northern Residents subtracted from that for the Southern Residents.

### Approval and ethics statement

DNA samples for this study are part of a long-term tissue collection not collected explicitly for this study. Therefore, this study did not require any interaction with live animals. All biopsies were collected under relevant guidelines and regulations set out by permits issued by Fisheries and Oceans Canada and/or the US National Marine Fisheries Service. Sample collection was carried out by a number of individuals representing multiple institutions. All biopsies were collected with permission and approval from their respective ethical committee or institutional review boards. The collection of most samples was performed under approval of the University of British Columbia and their Animal Care and Use Program.

## Supplementary Information

Supplementary Information.

## Data Availability

Code and methylation data are available at https://github.com/carlacrossman/StressMethylation.
