# Supplementary material for: An example of DNA methylation as a means to quantify stress in wildlife using killer whales
Source: Sci Rep. 2021 Aug 19;11:16822. doi: 10.1038/s41598-021-96255-1 (PMC8377091; doi:10.1038/s41598-021-96255-1)
Supplement: Supplementary file 1 — Supplementary Information. [file 41598_2021_96255_MOESM1_ESM.docx]

Supplementary Materials for

An example of DNA methylation as a means to quantify stress in wildlife using killer whales

Carla A. Crossman, Lance G. Barrett-Lennard, Timothy R. Frasier

Correspondence to: [crossman.ca@gmail.com](mailto:crossman.ca@gmail.com)

**This PDF file includes:**

Supplementary Methods Details

Fig S1. Percent Methylation Across Sites

Fig S2. CRF Promoter Region Sequence Alignment Across Species

Fig S3. Histograms of Read Depth at each CpG Site

Table S1. Primer Details

Table S2. Details of Samples Omitted Based on Read Depth

Supplementary Methods Details

Primer Design

We performed a literature search and identified genes involved in stress response that had demonstrated methylation changes in response to stressors in controlled studies. These included brain derived neurotropic factor (BDNF), corticotropin releasing factor (CRF) and glucocorticoid receptor (NR3C1) as well as a common control gene, β-actin (ACTB) whose methylation patterns should not be influenced by age, sex, or exposure to external stressors ^1^. We identified reference sequences for the promoter region of each of these genes from the published killer whale genome (GenBank accession GCA_000331955.1) using the provided annotations for the desired genes on NCBI Genome Browser.

We also confirmed the annotations by aligning the killer whale reference sequences to genes identified in a similar manner from species with better reference genomes (e.g. human, mouse, cow) Fig S1.

The sodium bisulfite treated DNA is single stranded and the strands are no-longer complimentary; therefore, a different primer pair is required to amplify each strand. We designed primer pairs for each strand. However, because the methylation patterns are complimentary on original strands, we only amplified samples using a single primer pair for each locus that showed the most reliable amplification (Table S1). Due to the low GC content in the DNA post-bisulfite conversion, long primers were needed to achieve the necessary specificity and annealing temperatures. Once we felt we had optimized conditions for each primer set, we performed Sanger sequencing to test that: (i) we had amplified the correct region, and (ii) to confirm successful conversion in the bisulfite treatment where the resulting sequences would be absent of cytosine except in potentially methylated sites.

Library Preparation

Following our initial PCR, we visualized a small amount of our products on 2% agarose gels. Using the remaining product, we performed a magnetic bead cleanup with Ampure XP beads using bead:sample ratios optimized for each primer set (ratios between 1.0:1 and 1.2:1) to eliminate all primer-dimers. In attempts to standardize the concentration of products across samples, the final elution volume at the end of the cleanup was adjusted based on the concentration determined from the pre-cleanup gel. (*i.e.* samples with concentrations <2.5ng/μl were eluted in 17.5μl, 2.5-10ng/μl were eluted in 27.5μl and >10ng/μl were eluted in 52.5μl.) The cleaned products were size-separated and visualized on another 2% agarose gel to confirm concentrations. We used 5μl of cleaned product in an indexing PCR to bind sample-specific index combinations and adapters for Illumina sequencing using the Nextera xt Indexes (Illumina).

We performed a final bead cleanup after the indexing PCR using bead:sample ratios between 0.8:1 and 1:1. Cleaned libraries were eluted in a final volume of 27.5μl of Tris-HCl except where product concentration following the first bead cleanup was still <2.5ng/μl in which case the elution volume was reduced to 17.5μl.

Processing Amplicon Sequences

In CUTADAPT 2.6 ^2^, we used an allowable error rate of 0.2 to separate loci and trim primer sequences. Our allowable error rate was higher than the default (0.1) to allow for more mistakes/mismatches within the primer sequence which CUTADAPT uses to identify distinct loci. Our main intention was to separate loci using the primer sequences and our primer sequences were sufficiently different from each other to allow this error rate. Trimming the primer sequences was less important for this study because they did not contain CpG sites that would be analyzed. We wanted to be more conservative in including reads at this stage as reads with poor alignment or poor quality would be filtered out later in the pipeline.

Bayesian Model

We built a Bayesian model using the percent methylation of each site as the predicted variable in a Bayesian regression analysis where individual, age, sex, population and CpG site were the predictor variables, as well as the interaction between specific CpG sites and population. We also allowed for different standard deviations in each population. We built similar models investigating other pairwise interactions and none had an effect on our results, so we left them out of our final model. The model was run with a combination of R and RStan with 2000 steps as the warm-up and 12000 steps with recorded data.

*mu =* *β_0_* + *β_1_*_[_*_i_*_]_*ind*_[_*_i_*_]_ + (*β_2_* * *age*) + *β_3_*_[_*_j_*_]_*sex*_[_*_j_*_]_ + *β_4_*_[_*_k_*_]_*site*_[_*_k_*_]_ + *β_5_*_[_*_l_*_]_*pop*_[_*_l_*_]_ + *β_6_*_[_*_k,l_*_]_*site*_[k]_*pop*_[_*_l_*_]_

*percent.methylation ~ normal*(*mu*, *sigma*_[_*_l_*_]_)

To aid model performance, the continuous variables (percent methylation and age) were standardized as a z-scores (by subtracting each value from the mean, then dividing by the standard deviation), which transformed each into a normal distribution with a mean of zero and a standard deviation of one. Therefore, the prior probabilities for β_0_ and β_2_ were normal distributions with a mean of zero and a standard deviation of one. The coefficients for the other (categorical) variables were estimated in a hierarchical manner, where the hyper-priors for each mean was a normal distribution with a mean of zero and a standard deviation of one, and the hyper-priors for each standard deviation was a Cauchy distribution with a location parameter of one and a scale parameter of one.

The model was run with a combination of R v. 3.6.0 and RStan. Performance of the MCMC process was assessed by examination of Rhat scores, effective sample size (ESS) estimates, and examination of trace plots. Performance of the model itself was tested using posterior predictive checks. The model was run with 2000 steps as the warm-up and 12000 steps with recorded data.

We reran the model under the same conditions omitting the sample that was collected from a freshly stranded carcass and this did not have an effect on our results.

1. Chen, I.-H. *et al.* Selection of reference genes for RT-qPCR studies in blood of beluga whales (Delphinapterus leucas). *PeerJ* **4**, e1810 (2016).

2. Martin, M. Cutadapt removes adapter sequences from high-throughput sequencing reads. *EMBnet.journal* **17**, 10–12 (2011).


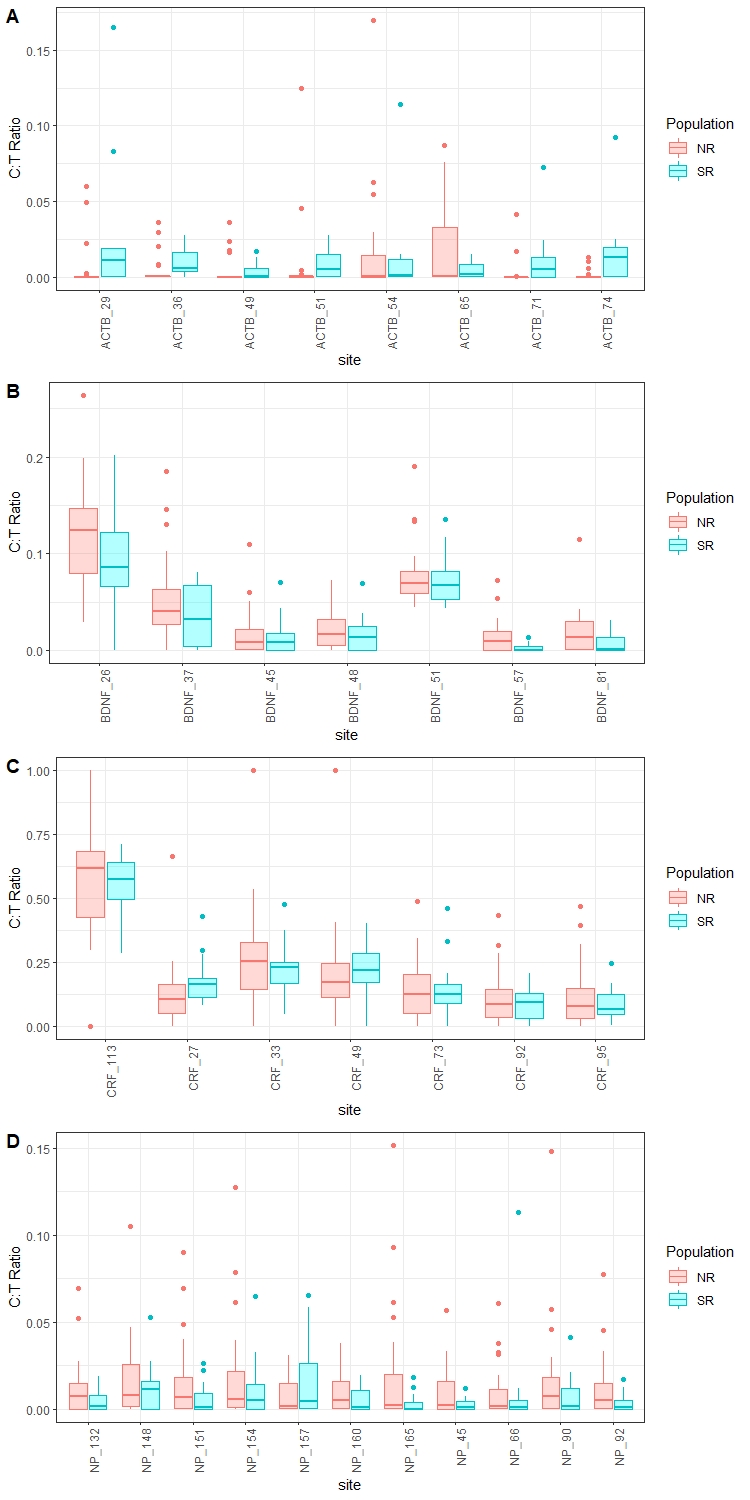
**Figure S1**. A comparison of the degree of methylation (C:T ratio in amplicon sequences – methylated : unmethylated bases) for CpG sites between populations for (A) ACTB, (B) BDNF, (C) CRF, (D) NR3C1. Northern Residents are represented in pink and Southern Residents in blue.

|  | 1 1 2 2 3 3 4 4 5 5 6 6  ----5----0----5----0----5----0----5----0----5----0----5----0----5 |
| --- | --- |
| Orcinus orca\|NW_004438481.1 Bos taurus\|NC_037341.1  Mus musculus\|NC_000069.7  Rattus norvegicus\|NC_005101.4  Homo sapiens\|NC_000008.11 | AGGAGCAGAGGCAGCA**CG**CAAT**CG**AGCTGTCAAGAGAG**CG**TCAGCTTATTAGGCAAATGCTG**CG**T  AGGAGCAGAGGCAGCGCGCAATCCAGCTGTCAAGAGAGCGTCAGCTTATTAGGCAAATGCTGCGT  AGGAGCAGAGGCAGCACGCAATCGAGCTGTCAAGAGAGCGTCAGCTTATTAGGCAAATGCTGCGT  AGGAGCAGAGGCAGCACGCAATCGAGCTGTCAAGAGAGCGTCAGCTTATTAGGCAAATGCTGCGT  AGGAGCAGAGGCAGCACGCAATCGAGCTGTCAAGAGAGCGTCAGCTTATTAGGCAAATGCTGCGT |
|  | 1 1 1 1 1  7 7 8 8 9 9 0 0 1 1 2  ----0----5----0----5----0----5----0----5----0----5----0-- |
| Orcinus orca\|NW_004438481.1 Bos taurus\|NC_037341.1  Mus musculus\|NC_000069.7  Rattus norvegicus\|NC_005101.4  Homo sapiens\|NC_000008.11 | GGTTTCTGAAGAGGGT**CG**A**CG**CTATAAAATCCCACTC**CG**GGCTCTGGTGTGGAGAAA  GGTTTCTGAAGAGGGTCGACACTATAAAATCCCCTTCCAGGCTCTGGTGTGGAGAAA  GCTTTCTGAAGAGGGTCGACATTATAAAATCTCACTCCAGGCTCTGGTGTGGAGAAA  GCTTTCTGAAGAGGGTCGACGTTATAAAATCTCACTCCGGGCTCTGGTGTGGAGAAA  GGTTTTTGAAGAGGGTCGACACTATAAAATCCCACTCCAGGCTCTGGAGTGGAGAAA |

**TSS**

**Figure S2.** Alignment of the CRF promoter region between the killer whale (*Orcinus orca*), cow (*Bos taurus*), mouse (*Mus musculus*), rat (*Rattus norvegicus*), human (*Homo sapiens*). Transcriptional start site (TSS) and CpG sites in the killer whale sequence (bold & underlined) are identified

**
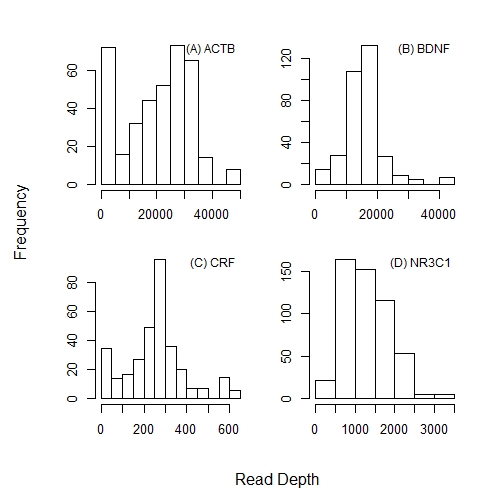
**

**Figure S3.** Histograms of read depth as calculated for each CpG site for (A) ACTB, (B) BDNF, (C) CRF and (D) NR3C1 used to determine threshold for omitting samples

Table S1.

Loci and primers used in this study.

| **Gene and Region** | **Abbr.** | **Locus-Specific Primer Sequence** | **Ta** | **Product Length** |
| --- | --- | --- | --- | --- |
| β-actin | ACTB | F: 5'-CTCTCTACCAATCCATCTCTC-3'  R: 5'-GAGTTATAAAAGGTAATTTTTGAA-3' | 58°C | 158bp |
| Brain Derived Neurotropic Factor | BDNF | F: 5'-TAAAGGAGTTATAATGAGTTGGT-3'  R: 5'-AACCCAACCTACACACTTACC-3' | 62°C | 276bp |
| Corticotropin Releasing Factor | CRF | F: 5'-TGATTTATGTAGGAGTAGAGG-3'  R: 5'-TAACAACTCAAACAATACAAAATTAAA-3' | 60°C | 351bp |
| Glucocorticoid Receptor | NR3C1 | F: 5'-TTTTTTTAAAAATAATAATTTAAAAATGT-3'  R: 5'-AAATTACAAAACAAAACCCACCCTC-3' | 58°C | 232bp |

**Table S2.**

Samples omitted from our model for insufficient read depth

| **Locus** | **Samples Omitted** | | | |
| --- | --- | --- | --- | --- |
|  | **N** | **Population** | **Sex** | **Age** |
| **ACTB** | 11 | SR  SR  SR  SR  SR  NR  NR  NR  NR  NR  NR | F  F  F  M  M  F  F  F  F  F  M | 38  23  26  22  19  21  26  24  19  26  6 |
| **BDNF** | 2 | SR  SR | M  F | 22  26 |
| **CRF** | 7 | SR  SR  SR  NR  NR  NR  NR | F  F  F  F  F  M  M | 38  26  30  26  17  38  20 |
| **NR3C1** | 2 | SR  SR | M  F | 22  26 |
